# Datasets for board meeting frequency and financial performance of Nigerian deposit money banks

**DOI:** 10.1016/j.dib.2018.06.044

**Published:** 2018-06-27

**Authors:** Damilola Felix Eluyela, Olamide Oluwabusola Akintimehin, Wisdom Okere, Emmanuel Ozordi, Godswill Osagie Osuma, Simon Osiregbemhe Ilogho, Olufemi Adebayo Oladipo

**Affiliations:** a Department of Accounting and Finance, Landmark University, Omu Aran, Nigeria; b Department of Business Studies, Landmark University, Omu Aran, Nigeria; c Department of Economics, Accounting and Finance, Bells University of Technology, Ota, Nigeria; d Department of Accounting, Covenant University, Ota, Nigeria; e Department of Banking and Finance, Covenant University, Ota, Nigeria

**Keywords:** Board meeting, Corporate governance, Deposit money banks, Firm performance

## Abstract

This article provides data on the impact of board meeting frequency and financial performance of deposit money banks in Nigeria. We obtained the dataset from Nigeria stock exchange (NSE) database. The time frame used for this work is 2010–2016. TOBIN Q was used as a major determinant of financial performance. The raw data is easily accessible on Nigeria stock exchange website. We describe the value of this data as well as the method to analyze the data.

**Specifications Table**

**Table:** 

Subject area	*Accounting, Management*
More specific subject area	*Corporate Governance*
Type of data	*Table, Excel File*
How data was acquired	*Collected from database of Nigerian stock exchange*
Data format	*Raw and analyzed*
Experimental factors	*Samples consisted of all deposit money banks listed on Nigeria stock exchange.*
Experimental features	*First descriptive statistics were provided followed by a correlation matrix. Then Hausman test were performed on the data to determine whether a fixed or random effect exist. Finally, panel regression analyses were performed.*
Data source location	*The data were obtained and collected from 14 deposit money banks listed on Nigeria stock exchange.*
Data accessibility	*The data are attached to this article.*

**Value of the data**

• The database provides the impact of board meeting frequency on financial performance of deposit money banks in Nigeria. To our knowledge, this is the complete dataset available for measuring how board meeting affect performance in Nigeria.
• The result provide empirical support for the agency theory, which suggest that when board meet more frequently, this will increase their ability to effectively monitor, advise, scrutinise and create an atmosphere of discipline.
• The dataset (financial performance variables) can be used by other researchers in carrying out subsequent research in area of other board attributes.

## 1 Data

The data includes the variables used to examine the impact of board meeting frequency on firm performance. 14 Deposit money banks listed on Nigeria stock exchange were used as the sample for the study. Time frame for this study was 2010–2016. Table 1 below shows data for the dependent and independent variable which are log of frequency of board meeting and the Tobin Q. Table 2 provides data for the control variables (board size and firm size). These are all processed data (except from board size). The raw data is attached to this article.

**Table 1:** Log of BMF and Tobin Q.

**DBM׳s**	**Log of BMF**							**TOB Q**						
	**1**	**2**	**3**	**4**	**5**	**6**	**7**	**1**	**2**	**3**	**4**	**5**	**6**	**7**
Access	0.845	0.903	0.903	0.845	0.845	0.778	1.000	1.212	1.208	1.072	1.133	1.132	1.146	1.136
Diamond	0.954	1.000	0.778	0.778	0.778	0.602	0.778	1.106	1.108	1.092	1.091	1.108	1.122	1.111
FCMB	0.845	0.903	0.778	0.477	0.778	0.699	0.778	1.196	1.195	1.145	1.143	1.137	1.140	1.153
Fidelity	0.845	0.903	0.845	0.699	1.079	1.000	0.954	1.202	1.198	1.177	1.151	1.146	1.149	1.143
First Bank	0.078	0.845	0.903	0.903	0.845	0.845	0.845	1.100	1.129	1.127	1.102	1.112	1.127	1.115
GTB	0.602	0.699	0.602	0.602	0.602	0699	0.699	1.138	1.154	1.177	1.170	1.169	1.178	1.162
Skye	0.903	0.903	0.903	0.845	0.845	1.000	0.954	1.110	1.110	1.097	1.109	1.102	1.087	1.087
Stanbic	0.477	0.602	0.301	0.602	0.699	0.699	0.602	1.172	1.153	1.127	1.133	1.128	1.138	1.134
Sterling	0.778	0.845	0.602	0.778	0.602	0.602	0.602	1.084	1.081	1.080	1.897	1.103	1.120	1.103
UBA	0.69	0.845	0.602	0.778	0.778	0.778	0.699	1.084	1.079	1.085	1.089	1.096	1.121	1.128
Union	0.778	0.845	0.778	0.778	0.699	0.845	0.903	1.191	1.189	1.188	1.199	1.220	1.235	1.217
Unity	0.602	0.602	0.778	1.114	0.845	0.954	1.000	1.113	1.118	1.130	1.070	1.185	1.186	1.175
Wema	0.778	0.778	0.699	0.699	0.602	0.602	0.602	1.026	1.028	1.005	1.125	1.114	1.116	1.114
Zenith	0.602	0.602	0.699	0.602	0.602	0.602	0.699	0.699	1.159	1.169	1.178	1.162	1.147	1.149

**Table 2:** Control variables.

**DBM׳s**	**Board size**							**Firm size**						
	**1**	**2**	**3**	**4**	**5**	**6**	**7**	**1**	**2**	**3**	**4**	**5**	**6**	**7**
Access	15	15	15	17	14	14	14	11.95	11.98	12.18	12.26	12.32	12.41	12.49
Diamond	19	19	15	18	15	16	11	11.88	11.90	12.07	12.18	12.29	12.24	12.31
FCMB	15	15	15	11	11	10	10	11.77	11.78	11.96	12.00	12.07	12.06	12.07
Fidelity	16	19	19	16	16	17	17	8.86	8.87	8.96	9.03	9.07	9.09	9.11
First Bank	11	12	19	8	13	13	13	9.54	9.45	9.50	9.57	9.62	9.60	9.65
GTB	13	14	14	14	14	16	16	12.16	12.18	12.21	12.28	12.38	12.36	12.49
Skye	11	12	14	13	13	17	17	8.96	8.96	9.03	9.05	9.14	9.08	9.08
Stanbic	12	12	12	11	7	7	7	8.65	8.74	8.83	8.88	8.97	8.97	9.02
Sterling	12	13	11	13	16	17	17	11.65	11.70	11.76	10.85	11.92	11.90	11.92
UBA	20	20	25	19	17	17	17	9.22	9.28	9.36	9.42	9.44	9.44	9.54
Union	12	14	14	16	14	13	13	9.01	9.02	9.01	9.00	9.00	9.02	9.10
Unity	15	17	17	15	14	15	15	11.56	11.57	11.60	11.61	11.62	11.65	11.68
Wema	10	10	12	12	13	14	14	11.32	11.34	11.39	11.52	11.58	11.60	11.63
Zenith	12	12	14	12	12	12	12	9.34	9.37	9.42	9.50	9.57	9.60	9.68

^***^Note: 1, 2, 3, 4, 5, 6 and 7 represent 2010, 2011, 2012, 2013, 2014, 2015 and 2016.

## 2 Experimental design, materials, and methods

We applied the following measurement to the raw data gathered from the annual reports of the listed 14 deposit money banks. Table 3 shows the measurement of variables.

**Table 3:** Measurement of variables.

**Variable name**	**Variable acronym**	**Variable type**	**Measurement**
Tobin Q	TOBQ	Dependent	Book value of total assets plus market value of equity minus book value of equity divided by book value of total assets
Board Meeting Frequency	BMF	Independent	Natural logarithm of a number of the board meeting held throughout the financial year
Board Size	BSize	Control	Total number of directors on the board
Firm Size	FSIZE	Control	Natural logarithm of total assets

We examined the relationship between board meeting frequency and financial performance of deposit money banks in Nigeria. To achieve this, we employed a panel methodology because the data applied for this study. Panel data is a combination of time series and cross sectional study [2]. One of the benefits of panel data is that it gives room for degrees of freedom and less collinearity among variables. The population for the purpose of this study consists of the fifteen (15) deposit money listed on the Nigerian Stock Exchange and we used sample size of 14. The sources of data include annual reports and accounts of selected deposit money banks. Prior to analyses, descriptive analysis were carried out to provide comprehensive information about the mean, minimum and maximum observations for each variable applied in this study. Correlation test were carried out to test multicollinearity and see the relationship between the dependent and independent variables. A multicollinearity exist when the correlation between two variables is greater than 0.8 [3], [4], [5]. Finally, the study carried out Hausman test to determine which model would be suitable for the panel regression: whether a fixed effect model or a random effect model [1], [4].
